# Point-of-care Ultrasound Diagnosis of an Atypical Acute Aortic Dissection

**DOI:** 10.5811/cpcem.2018.6.38106

**Published:** 2018-08-14

**Authors:** Richard Wroblewski, Ryan Gibbons, Thomas Costantino

**Affiliations:** * Temple University Hospital, Department of Emergency Medicine, Philadelphia, Pennsylvania; † Lewis Katz School of Medicine at Temple University, Department of Emergency Medicine, Philadelphia, Pennsylvania

## Abstract

Aortic dissections have a vast array of clinical presentations that rarely follow traditional teachings. Dissections are rapidly fatal conditions requiring immediate diagnosis and treatment to reduce morbidity and mortality. We present a case of an acute aortic dissection presenting as abrupt onset, atraumatic leg pain with absent distal extremity pulses. The prompt use of point-of-care ultrasound detected an intimal flap within the abdominal aorta allowing immediate surgical consultation and intervention.

## INTRODUCTION

The first documented case of an aortic dissection occurred in 1760 during the autopsy of King George II: “the pericardium was found distended with a quantity of coagulated blood… and in the trunk of the aorta we found a transverse fissure on its inner side.”^1^ Over 200 years later, our current understanding of acute aortic dissections is still evolving. The International Registry of Acute Aortic Dissection (IRAD), a large multicenter research consortium, has amassed a vast database providing physicians with extensive information regarding risk factors, examination findings, and historical clues that elicit further testing. Despite our present knowledge and technology, the overall in-hospital mortality is 27.4%.^2^ Diagnosing acute aortic dissections remains exceedingly difficult due to its varied presentations, unreliable physical exam findings, and relatively common risk factors associated with numerous other pathologies. We describe a case of an atypical presentation of an acute aortic dissection and the utility of point-of-care ultrasound (POCUS) in expediting the diagnosis and treatment.

## CASE PRESENTATION

A 67-year-old man presented to the emergency department (ED) complaining of sudden onset atraumatic right lower extremity pain shortly after using crack cocaine. He described the pain as sharp and burning throughout his entire leg. The patient denied chest pain, shortness of breath, abdominal pain, and focal neurologic symptoms. His past medical history was otherwise unremarkable.

On examination, the patient was alert and oriented but in obvious distress. Vital signs were within normal limits excluding a blood pressure of 180/100 millimeters of mercury (mmHg). The cardiopulmonary and abdominal examinations were benign with notably equal radial pulses. The patient’s right lower extremity was cool to touch without palpable pulses distal to and including the common femoral artery. The left lower extremity was warm with bounding pulses. On neurologic exam, the patient had normal and symmetric strength in his bilateral upper and lower extremities without apparent sensory deficits.

**Image 1:**
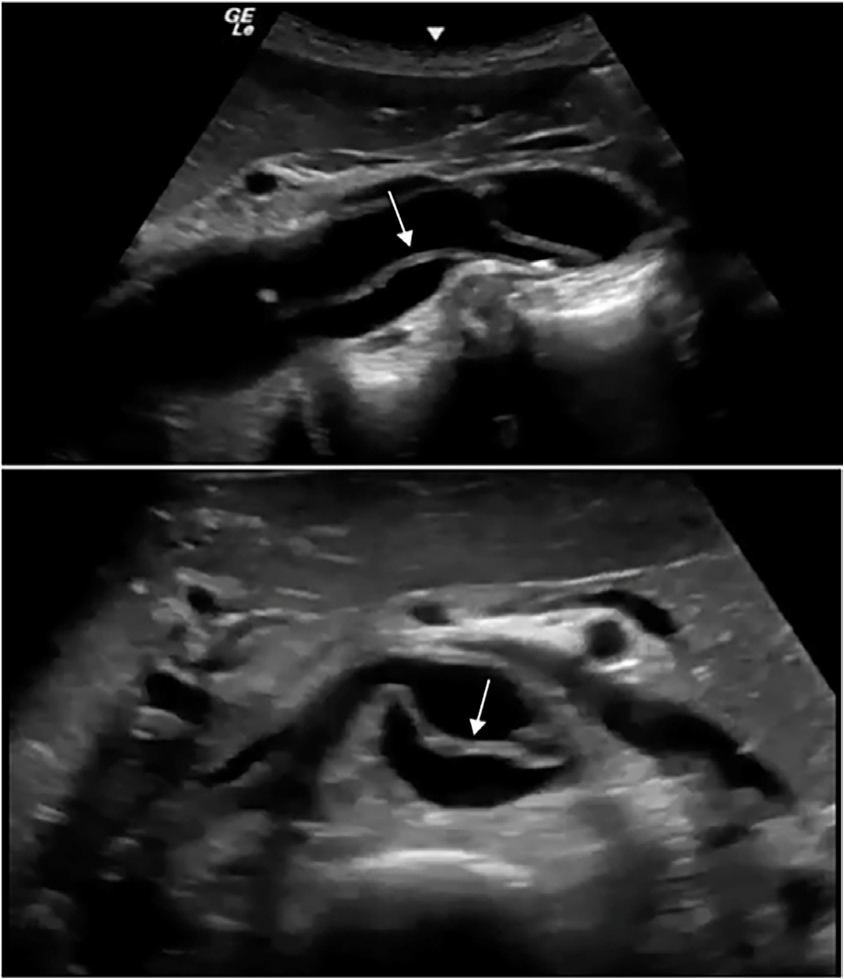
Sagittal (top) and transverse (bottom) ultrasonography view of abdominal aorta. Two views of the abdominal aorta. An arrow points to a large, undulating intimal flap noted within the abdominal aorta.

**Image 2:**
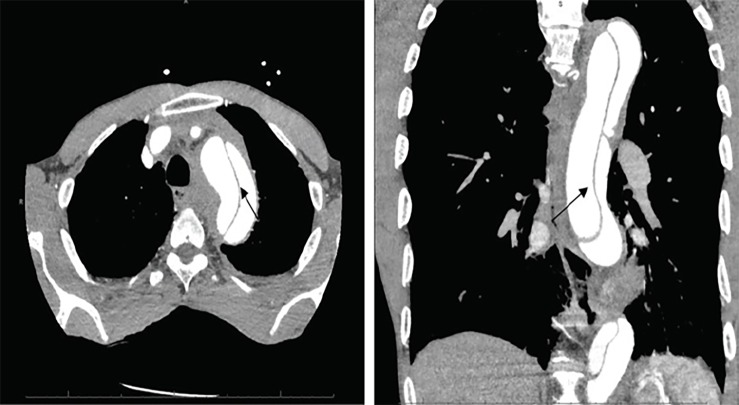
Transverse (left) and coronal (right) computed tomography imaging of the thoracic aorta. Images show a large aortic dissection extending distal from the left subclavian artery. An arrow points to the intimal flap that is visible within the aorta.

Based upon the patient’s acute presentation a POCUS was performed immediately in the ED. Our emergency ultrasound division has developed a protocol combining transthoracic echocardiography (TTE) and abdominal aorta ultrasound to evaluate for aortic pathology.^3^ POCUS demonstrated a large, undulating intimal flap within the abdominal aorta (Image 1). TTE did not reveal evidence of a Stanford Type A dissection. Immediate aggressive blood pressure control was initiated, and the patient was taken emergently for computed tomography (CT), which confirmed a Stanford Type B dissection (Image 2). Ultimately, the patient underwent thoracic endovascular aortic repair without complications.

## DISCUSSION

Characterized by an intimal tear in the aortic wall, an aortic dissection is a relatively rare entity with an annual incidence of nearly three per 100,000 patients.^4^ Even more challenging are the diverse clinical signs and symptoms encountered with dissections. The classic presentation of tearing, ripping chest pain is seen in <50% of patients.^2^ Furthermore, 10–15% of patients deny pain.^5^ The presentation of aortic dissection is so varied and difficult that Sullivan et al. found that emergency physicians evaluating confirmed cases only suspected aortic dissection in 43% of instances.^6^ In the absence of chest or abdominal pain, aortic dissection was not suspected.^6^

**CPC-EM Capsule**

What do we already know about this clinical entity?

Aortic Dissection is a rare disease with a high mortality rate. Diagnosis remains difficult to due to variances in how patients present to the emergency department.

What makes this presentation of disease reportable?

The rapid use of ultrasound to identify a highly specific finding for acute aortic dissection.

What is the major learning point?

Ultrasound can be used to aid in the rapid diagnosis of acute aortic dissection but is not sufficient to rule out the disease.

How might this improve emergency medicine practice?

It serves to remind us that use of point-of-care ultrasound is a rapid diagnostic tool and can be used to help expedite further work up to improve patient care.

IRAD has amassed a database to enhance our understanding of acute aortic dissections. Their findings highlight how unreliable history and physical are at diagnosing aortic dissections. Typical risk factors include male gender, age >50, connective tissue disorders, a family history of dissection, congenital aortic pathologies, trauma, and cocaine abuse.^2^ Chronic hypertension is the most common risk factor, yet systolic pressures above 150 mmHg are present in only 35.7% of Type A dissections and 70.1% of Type B dissections.^2^

Due to the wide variety of presentations for acute aortic dissections, most attempts at creating a clinical decision rule have failed. Rogers et al. attempted to create a simple clinical decision rule based on initial clinical suspicion as well as a quick, three-step bedside risk assessment. Patients at risk also had chest radiography and electrocardiograms. When the study was retrospectively applied to the IRAD it failed to identify nearly 5% of patients.^7^ The American College of Emergency Physicians (ACEP) evaluated this study, and other clinical decision rules, stating that the decision to work a patient up for acute aortic dissection should be based on physician discretion and that no evidence supported the routine use of clinical decision rules.^8^

One of the most significant impediments in diagnosing aortic dissections is limiting unnecessary advanced imaging without missing any presentations. Currently, ACEP recommends CT angiography as one of three gold-standard diagnostic imaging modalities for aortic dissection with a sensitivity above 98%.^8^ Magnetic resonance imaging and trans-esophageal echocardiography (TEE) are also considered gold standard, but limited availability precludes their routine utilization. More recent studies have analyzed laboratory values to enhance diagnostic ability. In particular, D-dimer values have been studied as a potential rule-out test but have not been validated.^8^

The use of POCUS in the ED for aortic pathology is increasing; however, there are limited data on its use for dissection. The majority of data regarding measurements and criteria for detection of aortic dissection is based on studies using TTE with which most emergency physicians have limited experience. The recent ACEP clinical policy reviewed six studies which evaluated the role of TTE in the diagnosis of aortic dissection. Each of the studies involved sonographer-performed or cardiologist-performed TTE, and their sensitivities and specificities varied significantly from 52%–80% and 0–100%, respectively.^7^ Most studies looked specifically for aortic root dilation, pericardial effusions, and recognition of an intimal flap; however, no set criteria were used for cutoff measurements. Although the presence of an intimal flap within the abdominal aorta has not been studied in isolation, Roudat et al. noted this finding to be 100% specific and 67% sensitive for aortic dissection. 9

Recent studies highlight the capability of emergency physician-performed POCUS to diagnose acute aortic dissections. An early case series published by Fojtik et al. initially underscored the ability of emergency providers to identify intra-aortic intimal flaps for the diagnosis of aortic dissections.^10^ A more recent prospective study published by Nazerian et al. demonstrated that emergency physicians using POCUS were 88% sensitive for Type A Stanford acute aortic dissections.^11^ When combined with a positive finding on their Aortic Dissection Detection risk score, their sensitivities improved to 96%.^11^ One of the most significant advantages of POCUS is its immediate availability. Pare et al. accentuated this fact, demonstrating a significant reduction in time to diagnosis of Sanford Type A dissections (>145 minutes) when employing emergency physician-performed sonography.^12^

Gibbons et al. developed an aortic dissection POCUS protocol combining TTE with abdominal aorta ultrasound.^3^ The protocol assessed for the presence of one of the following sonographic findings: pericardial effusion, intimal flap, or aortic outflow tract diameter measured at end-diastole >3.5cm.^3^ Their protocol identified 96.4% of patients with aortic dissections, confirmed on CT.^3^ Furthermore, their protocol was 100% sensitive for Sanford Type A dissection.^3^

Despite advances in diagnostic and treatment technology, the mortality of aortic dissection remains exceedingly high. Every hour delay in diagnosis results in a 1–2% increase in mortality. POCUS is becoming ubiquitous across emergency medicine, and it is a rapid, accurate means to screen for aortic dissections.

## CONCLUSION

Diagnosing an acute aortic dissection presents a unique challenge for all emergency providers. Its signs and symptoms lack sensitivity and specificity, and its exceedingly high mortality rate mandates prompt diagnosis and treatment. The role of point-of-care ultrasound continues to expand within the field of emergency medicine, and the aforementioned case and studies validate its efficacy to expedite the diagnosis and treatment of acute aortic pathology.

Documented patient informed consent and/or Institutional Review Board approval has been obtained and filed for publication of this case report.
